# Cardiac computed tomography in infective endocarditis: “bridging the detection gap”

**DOI:** 10.3389/fcvm.2024.1459833

**Published:** 2024-09-06

**Authors:** Natalie Montarello, Gabriel Bioh, Calum Byrne, Imtiaz Hassan, Vitaliy Androshchuk, Camelia Demetrescu, Sze Mun Mak, Ronak Rajani

**Affiliations:** ^1^ Cardiology Department, Guy's and St Thomas' NHS Foundation Trust, London, United Kingdom; ^2^ Department of Radiology, Guy’s and St Thomas’ NHS Foundation Trust, London, United Kingdom; ^3^ School of Biomedical Engineering and Imaging Sciences, Faculty of Life Sciences & Medicine, King’s College London, London, United Kingdom

**Keywords:** infective endocarditis, Duke criteria, cardiac computed tomography (CT) imaging, vegetations, pseudoaneursym

## Abstract

Infective Endocarditis (IE) remains a significant health challenge. Despite an increasing awareness, mortality is high and has remained largely unchanged over recent decades. Early diagnosis of IE is imperative and to assist clinicians several diagnostic criteria have been proposed. The best known are the Duke criteria. Originally published in 1994, these criteria have undergone significant modifications. This manuscript provides a timeline of the successive changes that have been made over the last 30 years. Changes which to a large degree have reflected both the evolving epidemiology of IE and the proliferation and increasing availability of advanced multi-modality imaging. Importantly, many of these changes now form part of societal guidelines for the diagnosis of IE. To provide validation for the incorporation of cardiac computed tomography (CT) in current guidelines, the manuscript demonstrates a spectrum of pictorial case studies that re-enforce the utility and growing importance of early cardiac CT in the diagnosis and treatment of suspected IE.

## Introduction

Infective endocarditis (IE) remains a significant health challenge. It has an annual incidence of 3–10/100,000 of the population and carries a mortality rate of up to 30% at 30 days (1). This has necessitated better means for establishing an early diagnosis of IE to enable the administration of prompt antimicrobial therapy, prevent intracardiac damage and enable earlier guideline-based referrals for surgery. Current diagnostic algorithms begin with a careful clinical history and examination. This is followed by microbiological evaluation of at least three sets of blood cultures, and an imaging assessment to determine the presence of endocardial tissue involvement. Given the protean nature of IE, several criteria have been proposed to aid clinicians establish a diagnosis of IE. The most well-known of these are the Duke Criteria for the diagnosis of IE. These were originally published in 1994 (2) and thereafter modified in 2000 (3). With a change in the epidemiology of IE, increasing use of intracardiac electronic devices (ICEDs), and the emergence of newer risk factors, further changes to the Modified Duke Criteria were thereafter proposed in 2015 by the European Society of Cardiology (ESC) (4) and then again by the International Society for Cardiovascular Infectious Diseases/ESC in 2023 (5, 6).

The original 1994 Duke criteria was comprised of both major and minor criteria for endocarditis (2). The first major criterion was the presence of persistently positive blood cultures with organisms typical for IE (*Viridans streptococci*, *Streptococcus bovis*, HACEK group, community acquired *Staphylococcus aureus* or enterococci in the absence of a primary focus). The second major criterion was evidence of endocardial involvement with echocardiography. This was defined as an oscillating mass on a valve or its supporting structures, an abscess or new dehiscence of a prosthetic valve or new valvular regurgitation. Minor criteria included a predisposition to IE, a fever ≥38°C, vascular phenomena (emboli, haemorrhage, Janeway lesions), immunologic phenomena (glomerulonephritis, Osler's nodes, Roth Spots, rheumatoid factor), microbiological evidence not meeting major criterion, and echocardiography consistent with IE but not meeting major criterion. With these a diagnosis of definitive IE could be made in the presence of 2 major, 1 major and 3 minor, or 5 minor criteria. Endocarditis was rejected however in the presence of a firm alternative diagnosis, resolution of the manifestations of IE within antibiotic therapy for 4 days or less or when there was no pathological evidence of IE after 4 days or less of antibiotic therapy. Possible IE was considered for cases that did not fall in either these two categories. Following the release of the original Duke criteria, validation studies confirmed a high sensitivity and specificity for the criteria and the diagnostic utility of echocardiography in confirming definite endocarditis (7, 8).

In 2000 the Duke criteria were modified to include *Staphylococcus aureus* bacteraemia as a major criterion irrespective of whether it was hospital or community acquired. This reflected more contemporary studies documenting IE in patients with nosocomial staphylococcal bacteraemia. Furthermore, serological evidence for *Coxiella burnetti* or anti-phase 1 IgG antibody titre ≥1:800 was moved from a minor criterion to a major one. The minor criterion of an “echocardiogram being consistent with IE but not meeting major criterion” was also removed owing to its rare usage. Transoesophageal echocardiography (TOE) was recommended in all patients with possible prosthetic valve endocarditis (PVE) and transthoracic echocardiography (TTE) as a first line test in all other patients. Finally, there was an adjustment of the Duke criteria to require a minimum of 1 major plus 1 minor or 3 minor criteria to designate possible IE (3).

The ESC thereafter amended the modified Duke criteria in 2015 to reflect the evolving epidemiology of IE. Although the criteria performed well in patients with native valve disease, their performance in patients with ICEDs and prosthetic heart valves (PHVs) was less optimal. With rapid developments in metabolic imaging with positron emission tomography (PET) and cardiac computed tomography (CT), their role in the diagnosis and management of IE had become more established and were thus incorporated. Abnormal activity around the site of PHV implantation detected by ^18^F-Fluorodeoxyglucose Positron Emission Tomography/Computed Tomography (^18^F-FDG PET/CT) or radiolabelled leucocyte single-photon emission computed tomography (WBC SPECT/CT) at >3 months was now included as a major imaging criterion as was the detection of paravalvular lesions on cardiac CT (4).

In 2023 the International Society for Cardiovascular Infectious Diseases further updated the modified Duke criteria (5) (Table 1). As well as some notable changes to the standard microbiological criteria, there were developments with the imaging criteria and more refinement to the minor criteria. These changes reflected advancements in microbiological diagnostic technique, epidemiological trends and the proliferation and more widespread access to advanced imaging. From a cardiac imaging perspective more recognition was given to the spectrum of cardiac changes that are associated with IE (vegetations, valve perforation, pseudoaneurysm, abscess formation, fistula formation and valvular dehiscence) and to the optimal techniques to detect these. Transthoracic echocardiography remained as a first line test with the threshold for TOE being lowered for patients with PHVs or ICEDs. Cardiac CT was also added as an additional complimentary imaging modality owing to its superior ability to TOE to detect paravalvular complications, particularly in patients with PHVs or ICEDs (9). ^18^F-Fluorodeoxyglucose Positron Emission Tomography/Computed Tomography was also added as an imaging modality owing to its ability to reclassify a large proportion of patients with PVE from “possible” to “definite” IE with a sensitivity of 86% and specificity of 84% (10). In addition, there was an introduction of the radiological detection of arterial emboli, septic emboli, cerebral or splenic abscesses, mycotic aneurysms and intracranial haemorrhage to the minor criterion of “vascular phenomena”. The ESC latest guidelines for IE in late 2023 incorporated these changes with imaging evidence for IE including echocardiography, cardiac CT, ^18^F-FDG PET/CT and WBC SPECT/CT all qualifying as a major criterion for IE. Cardiac CT now received a Class 1B recommendation for patients with suspected native valve endocarditis (NVE) and PVE to detect valvular and paravalvular lesions, and to confirm the diagnosis of IE. Whole body CT similarly received a class 1B recommendation in symptomatic patients with NVE and PVE to detect peripheral embolism or add to the minor diagnostic criteria (6).

**Table 1:** Current modified Duke criteria for infective endocarditis (2023) (5).

I. Major criteria
A. Microbiologic major criteria 1. Positive blood cultures i. Microorganisms that commonly cause IE isolated from 2 or more separate blood culture sets (Typical), or ii. Microorganisms that occasionally or rarely cause IE isolated from 3 or more separate blood culture sets (Non-typical) 2. Positive laboratory tests i. Positive polymerase chain reaction (PCR) or other nucleic acid-based technique for *Coxiella burnetii*^a^*, Bartonella* species, or *Tropheryma whipplei* from blood, or ii. *Coxiella burnetii* antiphase 1 immunoglobulin G (IgG) antibody titre >1:800, or isolated from a single blood culture^a^, or iii. Indirect immunofluorescence assays (IFA) for detection of IgM and IgG antibodies to *Bartonella henselae* or *Bartonella quintana* with immunoglobulin G (IgG) titre ≥1:800
B. Imaging major criteria 1. Echocardiography and cardiac computed tomography (CT) imaging i. Echocardiography and/or cardiac CT^b^^,^^c^ showing vegetation, valvular/leaflet perforation, valvular/leaflet aneurysm, abscess, pseudoaneurysm, or intracardiac fistula, or ii. Significant new valvular regurgitation on echocardiography as compared with previous imaging. Worsening or changing of preexisting regurgitation is not sufficient, or iii. New partial dehiscence of prosthetic valve as compared with previous imaging 2. Positron emission computed tomography with ^18^F-Fluorodeoxyglucose (^18^F-FDG PET/CT imaging)^b^^,^^c^ Abnormal metabolic activity involving a native or prosthetic valve, ascending aortic graft (with concomitant evidence of valve involvement), intracardiac device leads or other prosthetic material
C. Surgical major criteria 1. Evidence of IE documented by direct inspection during heart surgery neither Major Imaging Criteria nor subsequent histologic or microbiologic confirmation
II. Minor criteria
A. Predisposition - Previous history of IE - Prosthetic valve - Previous valve repair - Congenital heart disease - More than mild regurgitation or stenosis of any aetiology - Endovascular intracardiac implantable electronic device (CIED) - Hypertrophic obstructive cardiomyopathy - Injection drug use B. Fever: Documented temperature greater than 38°C (100.4°F) C. Vascular Phenomena: Clinical or radiological evidence of arterial emboli, septic pulmonary infarcts, cerebral or splenic abscess, mycotic aneurysm, intracranial haemorrhage, conjunctival haemorrhages, Janeway lesions, purulent purpura^c^ D. Immunologic Phenomena: Positive rheumatoid factor, Osler nodes, Roth spots, or immune complex-mediated glomerulonephritis E. Microbiologic evidence, falling short of a major criterion 1. Positive blood cultures for a microorganism consistent with IE but not meeting the requirements for Major Criterion, or 2. Positive culture, PCR, or other nucleic acid based test for an organism consistent with IE from a sterile body site other than cardiac tissue, cardiac prosthesis, or arterial embolus; or a single finding of a skin bacterium by PCR on a valve or wire without additional clinical or microbiological supporting evidence F. Imaging criteria Abnormal metabolic activity as detected by ^18^F-FDG PET/CT within 3 months of implantation of prosthetic valve, ascending aortic graft (with concomitant evidence of valve involvement), intracardiac device leads or other prosthetic material G. Physical examination criteria New valvular regurgitation identified on auscultation if echocardiography is not available. Worsening or changing of preexisting murmur not sufficient
I. Definite endocarditis
A. Pathologic criteria 1. Microorganisms identified in the context of clinical signs of active endocarditis in a vegetation; from cardiac tissue; from an explanted prosthetic valve or sewing ring; from an ascending aortic graft (with concomitant evidence of valve involvement); from an endovascular intracardiac implantable electronic device (CIED); or from an arterial embolus, or 2. Active endocarditis (may be acute or subacute/chronic) identified in or on a vegetation; from cardiac tissue; from an explanted prosthetic valve or sewing ring; from an ascending aortic graft (with concomitant evidence of valve involvement); from a CIED; or from an arterial embolus B. Clinical criteria 1. 2 major criteria, or 2. 1 major criterion and 3 minor criteria, or 3. 5 minor criteria
II. Possible endocarditis
A. 1 major criterion and 1 minor criterion^a^, or B. 3 minor criteria^a^
III. Rejected endocarditis
A. Firm alternate diagnosis explaining signs/symptoms, or B. Lack of recurrence despite antibiotic therapy for less than 4 days, or C. CNo pathologic or macroscopic evidence of IE at surgery or autopsy, with antibiotic therapy for less than 4 days, or D. Does not meet criteria for possible IE, as above

Duke diagnostic criteria:

^a^ 2000 modifications.

^b^ 2015 modifications, included incorporating positive ^18^F-FDG PET/CT as a major criteria.

^c^ 2023 modifications, included increasing emphasis on cardiac CT (ability to detect paravalvular complications in PHVs or ICEDs) and ^18^F-FDG PET/CT (ability to reclassify “possible” to “definite” IE) as imaging modalities.

Although it is envisaged that these new changes will improve the sensitivity and maintain the specificity for the diagnosis of IE, the original Duke and subsequent Modified Duke Criteria only received subsequent external validation a few years following their introduction. The 1994 Duke Criteria and subsequent revised Criteria in 2000 were shown to have a sensitivity of approximately 80% for the detection of IE (11). Despite this, they were shown to be less effective in patients with PHVs and ICEDs. This resulted in the incorporation of ^18^F-FDG PET/CT for PVE as a major imaging criterion in the ESC 2015 Duke Criteria. With the latest 2023 ESC Duke Criteria Guidelines there are only few validation studies that detail the value of these changes in the determination of “definite” IE.

The aim of the current pictorial review is to firstly present several clinical vignettes from a large IE specialist centre in the United Kingdom that has utilised systematic cardiac CT for the diagnosis and management of IE over the last 4 years. We furthermore also detail our personal experience on the strengths of this technique in this setting.

## Methods

**Figure 1:**
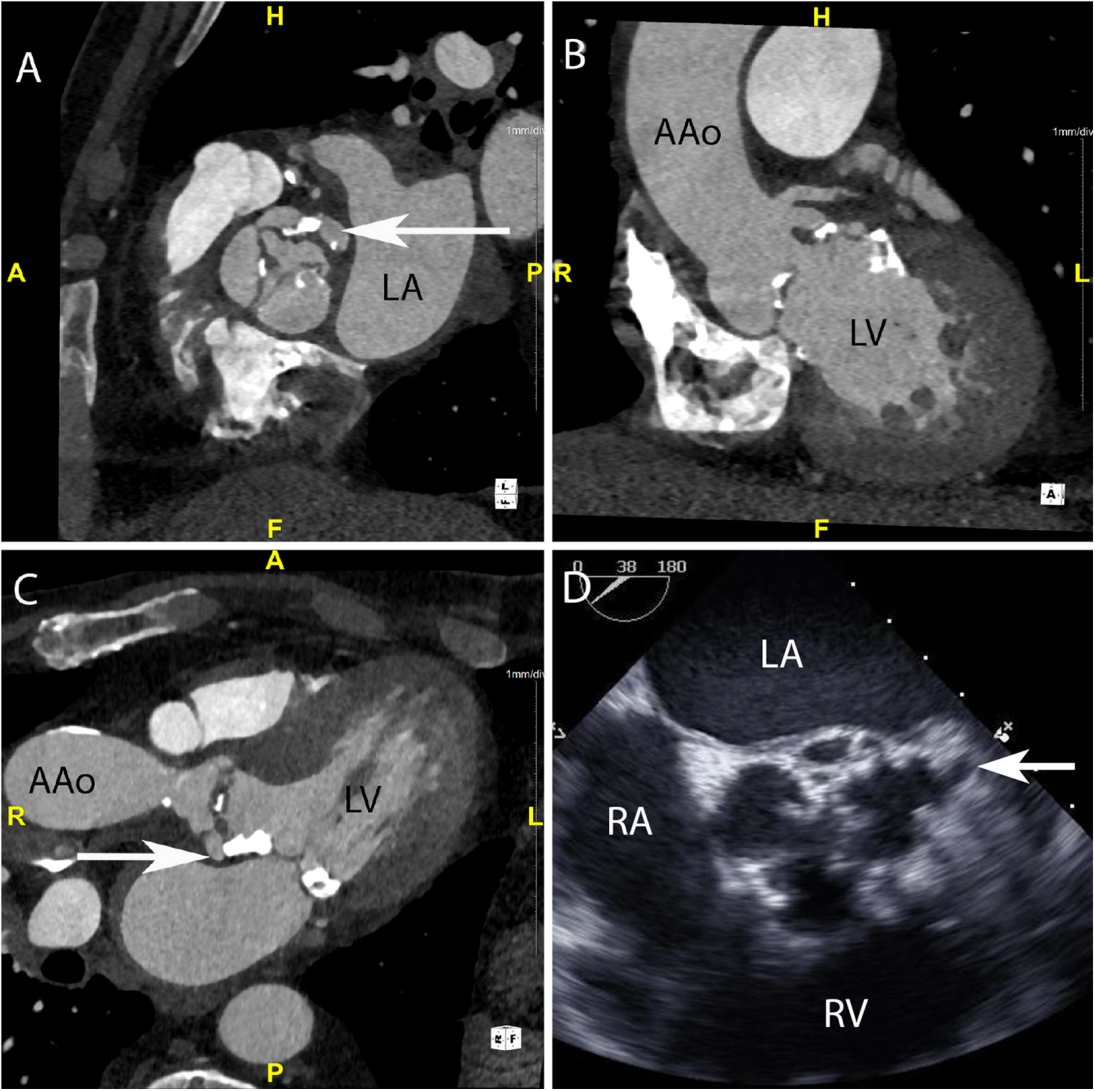
CASE 1 - A 75-year-old man presented feeling generally unwell with fever. *Staphylococcus aureus* was isolated from blood cultures (see text). LA, left atrium; LV, left ventricle; RA, right atrium; RV, right ventricle; AAo, ascending aorta.

**Figure 2:**
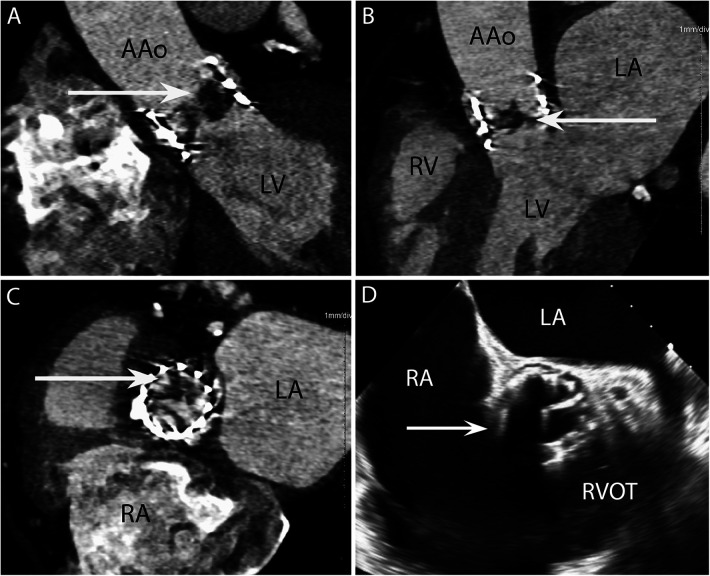
CASE 2 - A 77-year-old man with a history of transcatheter aortic valve implantation (TAVI) presented with a sore throat, diarrhoea, and a temperature of 40°C. *Staphylococcus aureus* was isolated from blood cultures (see text). RA, right atrium; RVOT, right ventricular outflow tract; RV, right ventricle; LA, left atrium; LV, left ventricle; AAo, ascending aorta.

**Figure 3:**
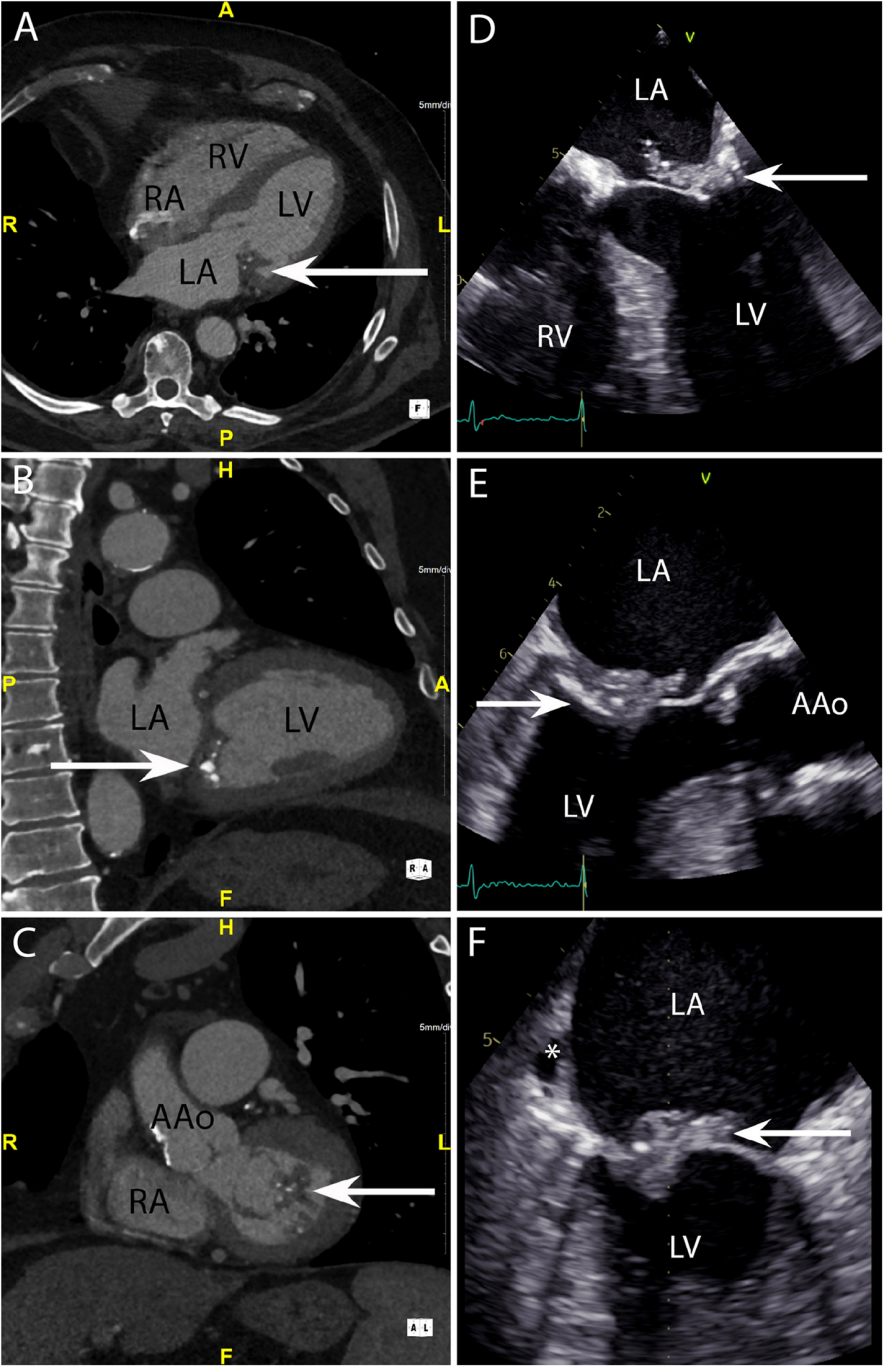
CASE 3 - A 79-year-old man presented with shortness of breath and fever. Blood cultures grew *Staphylococcus aureus* (see text). RA, right atrium; RV, right ventricle; LA, left atrium; LV, left ventricle; AAo, ascending aorta.

**Figure 4:**
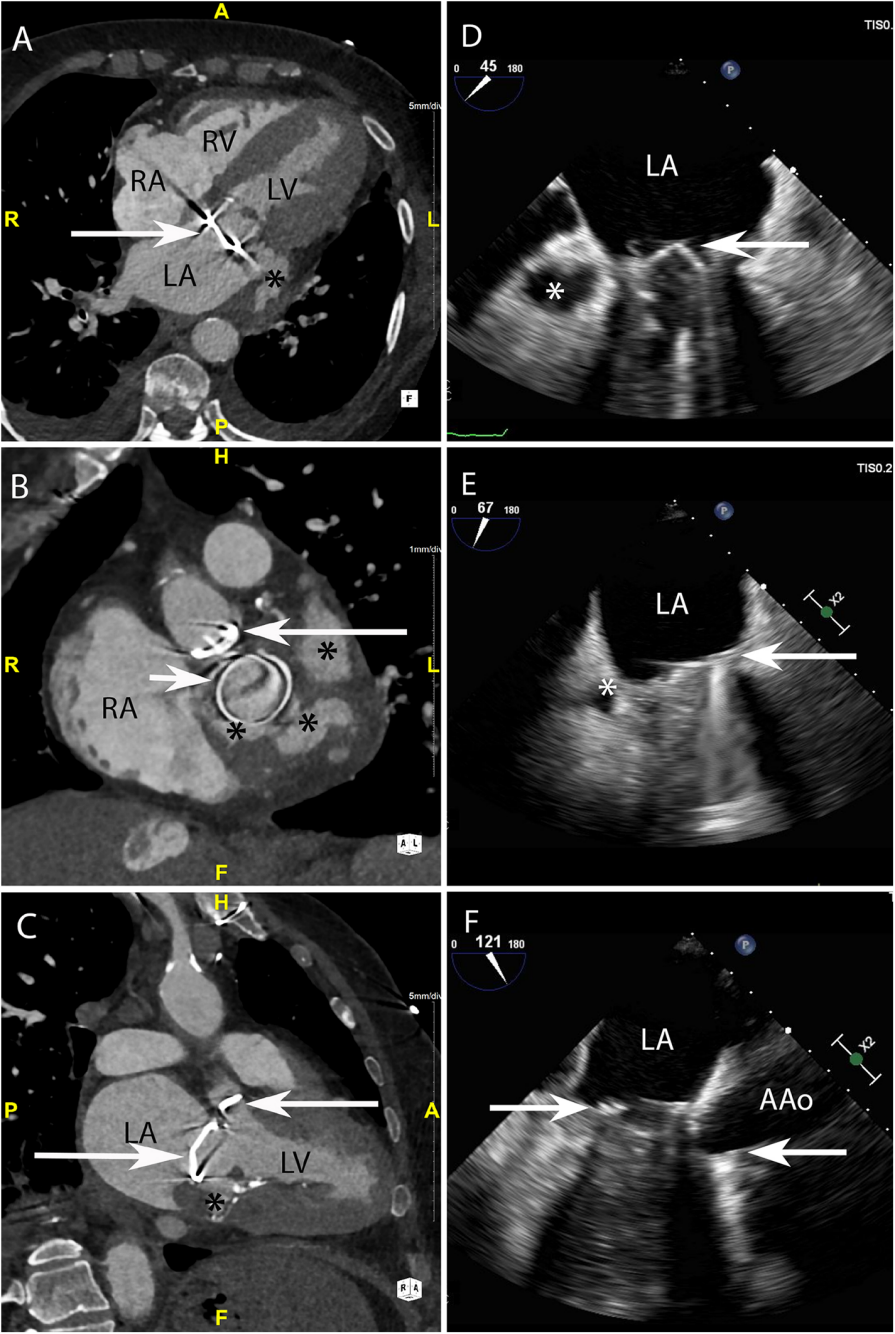
CASE 4 - A 79-year-old man with metallic mitral and aortic valve prostheses undergoing treatment for IE remained febrile and unwell despite appropriate guideline recommended antibiotic therapy (see text). RA, right atrium; RV, right ventricle; LA, left atrium; LV, left ventricle; AAo, ascending aorta.

**Figure 5:**
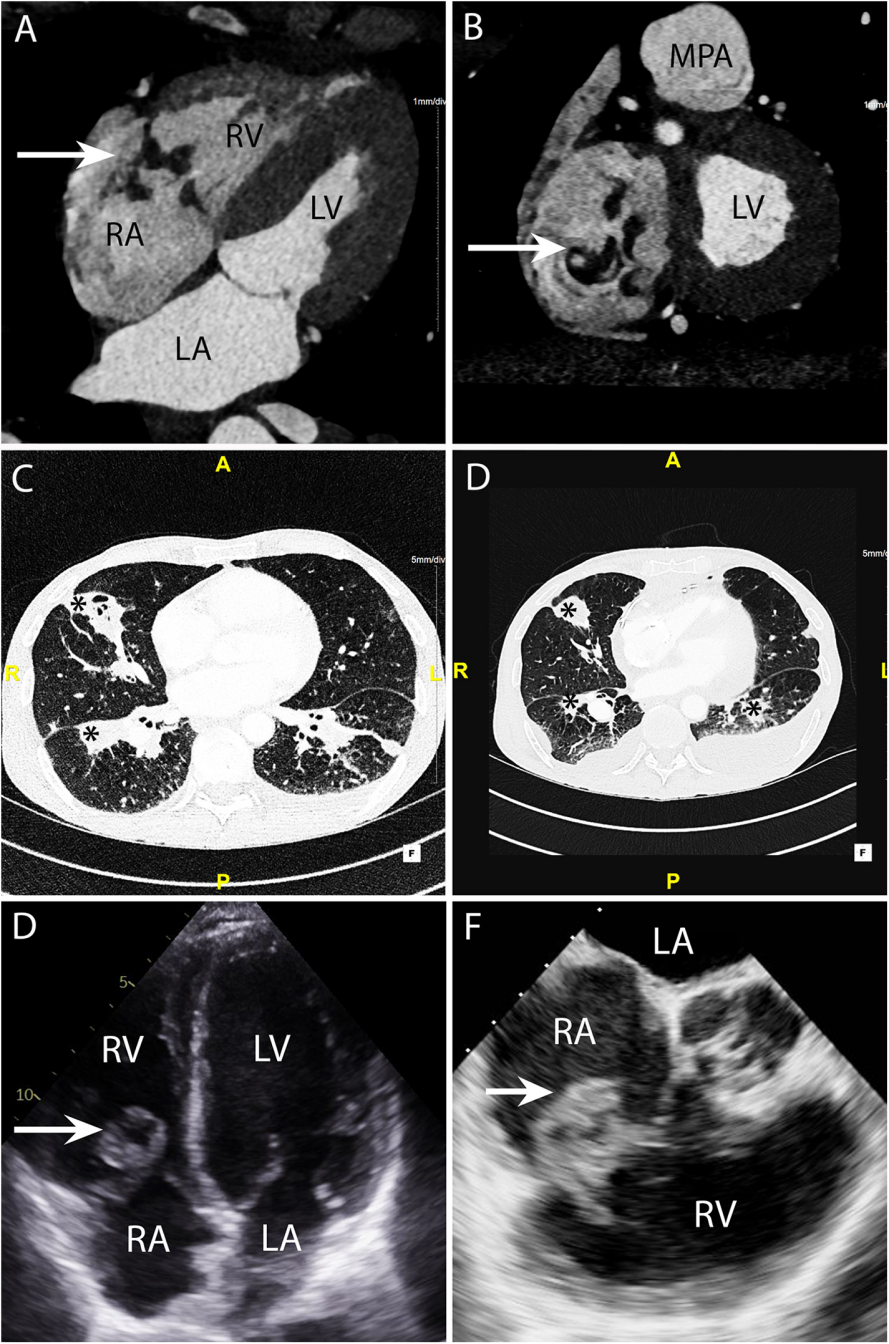
CASE 5 - A 53-year-old man presented with fever, dyspnoea and confusion on a backgound history of intravenous drug use. Blood cultures grew *Staphylococcus aureus* (see text). RA, right atrium; RV, right ventricle; MPA, mean pulmonary artery; LA, left atrium; LV, left ventricle.

**Figure 6:**
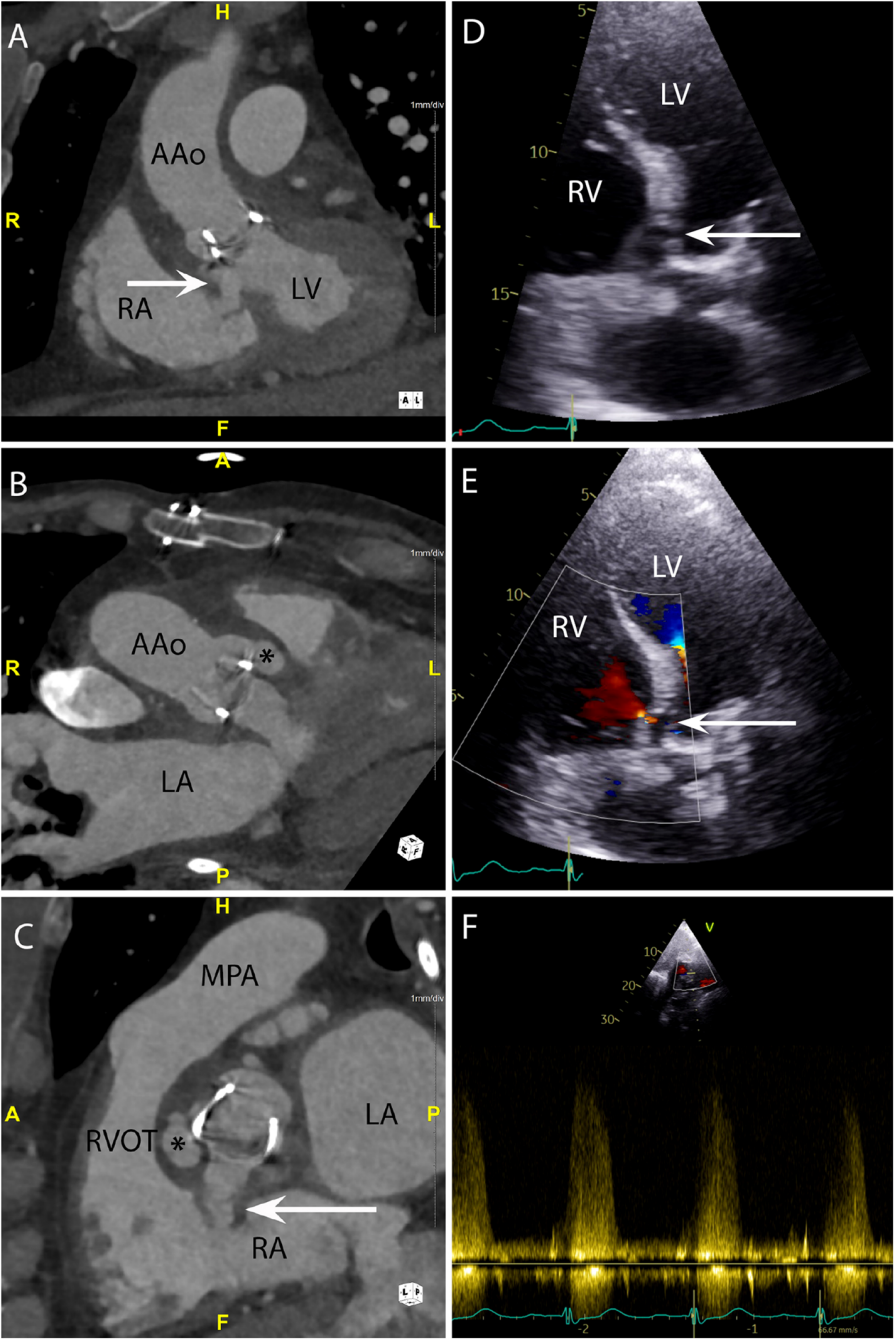
CASE 6 - A 71-year-old man presented with fever, confusion and fatigue. *Enterococcus faecalis* was isolated from blood cultures (see text). RA, right atrium; RVOT, right ventricular outflow tract; RV, right ventricle; MPA, mean pulmonary artery; LA, left atrium; LV, left ventricle; AAo, ascending aorta.

**Figure 7:**
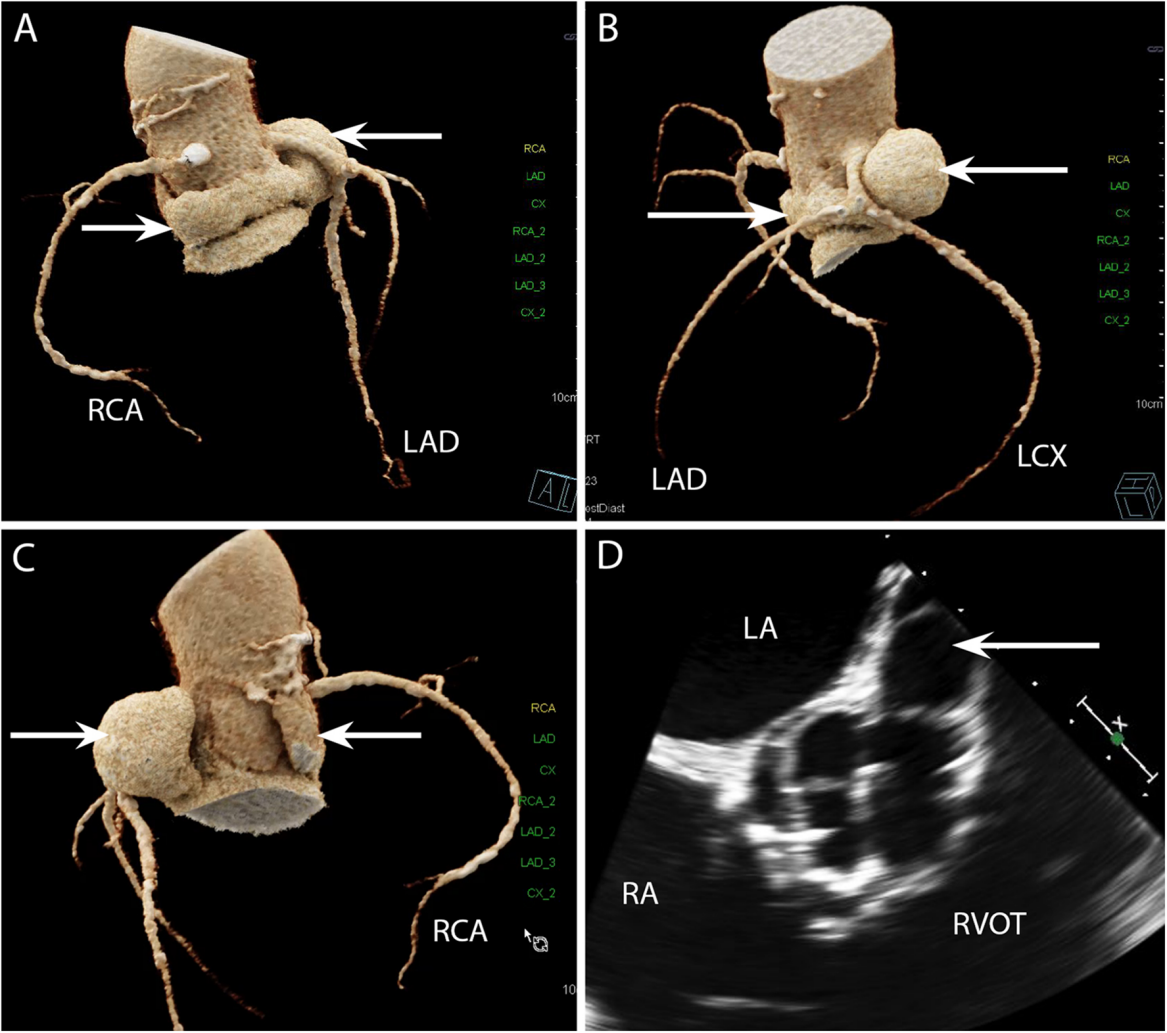
CASE 7 - A 69-year-old man presented with decompensated heart failure. Blood cultures were negative (see text). LAD, left anterior descending artery; LCX, left circumflex artery; RCA, right coronary artery; LA, left atrium; RA, right atrium; RVOT, right ventricular outflow tract.

Guy's and St Thomas NHS Foundation Trust is a large tertiary cardiac centre in London that provides specialist IE services to the South London and Kent network. It currently receives 100–130 new cases of IE per annum. The department performs approximately 25,000 transthoracic echocardiograms, 1,000 transoesophageal echocardiograms, 6,000 cardiac magnetic resonance (CMR) scans and 2,500 cardiac CT scans per annum. Access to cardiac CT for IE is normally on the same day for urgent cases, with PET imaging being available but less accessible owing to pressures on cancer imaging. In keeping with the 2015 ESC guidelines, our principal imaging modalities for IE detection have been TTE and TOE. With the COVID pandemic and the reduction of TOE utilisation, from 2020 we utilised cardiac CT at an ever-increasing rate with it now becoming an almost universal imaging modality for our IE patient cohort. For the purposes of the current pictorial review, cases were selected specifically to highlight the imaging appearances of IE on cardiac CT across both NV and PHV endocarditis (Cases/Figures 1–7).

### Case 1

A 75-year-old man presented feeling generally unwell with fever. *Staphylococcus aureus* was isolated from blood cultures. Cardiac CT was performed: An in-plane view (Figure 1A) of the aortic valve demonstrated a low-density soft tissue vegetation on all three cusps and a pseudoaneurysm (arrow) which communicated with the left coronary sinus (HU 420). Oblique coronal (Figure 1B) and axial (Figure 1C) views demonstrated that the vegetation was attached to both sides of the aortic valve cusps and showed the presence of a small pseudoaneurysm (arrow) that had been missed on TTE. *Although aortic root abscesses and pseudoaneurysms are often used interchangeably in cardiac imaging, on CT a pseudoaneurysm is defined as a perivalvular cavity that is in communication with either a heart chamber or major blood vessel resulting from rupture of an abscess into that cavity. In contrast, an abscess is a closed cavity with necrosis and purulent material which appears as a perivalvular collection(s) of fluid encased in a thick layer of inflammatory tissue enhanced by the injection of contrast medium*. Preceding TOE images (Figure 1D) taken in the mid-oesophageal aortic valve short axis view (ME AV SAX view) failed to accurately differentiate aortic valve calcification from small vegetations. There was a presumed small pseudoaneurysm communicating with the left coronary cusp (arrow) but its extent could not be determined.

### Case 2

A 77-year-old man with a history of transcatheter aortic valve implantation (TAVI) presented with a sore throat, diarrhoea, and a temperature of 40°C. *Staphylococcus aureus* was isolated from blood cultures. Cardiac CT was performed within 24 h. The oblique coronal (Figure 2A) and 3-chamber (Figure 2B) views demonstrated a low-density soft tissue vegetation (arrow) with a Hounsfield Unit (HU) value 80 attached to the TAVI leaflets. An in-plane view (Figure 2C) showed that the vegetation (arrow) involved all three cusps of the implanted valve. In the initial TOE images (Figure 2D) taken in the ME AV SAX view, the TAVI nitinol stent frame obscured adequate prosthetic valve leaflet evaluation (arrow) and aortic root involvement could not be established.

### Case 3

A 79-year-old man presented with shortness of breath and fever. Blood cultures grew *Staphylococcus aureus*. The axial 4-chamber (Figure 3A) and oblique sagittal 2-chamber (Figure 3B) views demonstrated a low-density soft tissue (HU 78) vegetation (arrow) attached to a calcified posterior mitral valve leaflet. An oblique coronal view (Figure 3C) confirmed these findings and highlighted possible involvement of the anterior leaflet. Preceding TOE images in the mid-oesophageal (ME) 4-chamber (Figure 3D), ME 2-chamber (Figure 3E) and ME 3-chamber (Figure 3F) views demonstrated a large heterogenous calcified mass attached to the posterior mitral valve leaflet (arrows) consistent with a vegetation. A possible paravalvular pseudoaneurysm with disruption at the LA free wall (asterisk) was noted but was excluded on subsequent CT.

### Case 4

A 79-year-old man with metallic mitral and aortic valve prostheses (arrows) undergoing treatment for IE remained febrile and unwell despite appropriate guideline recommended antibiotic therapy. Blood cultures remained positive for *Staphylococcus aureus*. Cardiac CT was performed: Axial (Figure 4A) and in-plane (Figure 4B) views demonstrated a multi-lobulated thick walled paravalvular pseudoaneurysm (asterisks) (HU 231) which extended posteriorly and laterally to the sewing ring of the valve prosthesis. Oblique sagittal 2-chamber (Figure 4C) demonstrated additional inferior extension of the pseudoaneurysm (asterisk). Preceding TOE images taken from the ME mitral commissural (Figure 4D), ME 2-chamber (Figure 4E) and ME 3-chamber (Figure 4F) views showed a likely paravalvular pseudoaneursym (asterisk) but its extent or size could not be adequatly determined leading to a CT being performed.

### Case 5

A 53-year-old man presented with fever, dyspnoea and confusion on a backgound history of intravenous drug use. Blood cultures grew *Staphylococcus aureus*. CT brain imaging showed a subarachnoid haemorrhage. Cardiac CT was performed: Axial 4-chamber (Figure 5A) and oblique coronal (Figure 5B) views showed a low density soft tissue vegetation (arrow) (HU 59) attached to the tricuspid valve leaflets with an associated perforation (arrow) over the posterior tricuspid valve leaflet (Figure 5B). The wide field of view reconstructions (Figures 5C and 5D) showed bilateral foci of cavitating consolidation consistent with septic emboli (asterisks). Prior to CT, a TTE and TOE were performed. TTE 4-chamber (Figure 5E) and TOE ME AV SAX (Figure 5F) views demonstrated a large mobile heterogenous mass consistent with a large vegetation. A cardiac CT and a flash ECG-gated aorta were arranged to further elucidate leaflet morphology, assess for extra-cardiac complications and evaluate the coronary arteries prior to surgical intervention.

### Case 6

A 71-year-old man presented with fever, confusion and fatigue. *Enterococcus faecalis* was isolated from blood cultures*.* Cardiac CT was performed: An oblique coronal (Figure 6A) view demonstrated a paravalvular fistula (arrow) extending from the region of the aortic valve non-coronary cusp (NCC) and left ventricular outflow tract into the right atrium (RA). An oblique axial view (Figure 6B) demonstrated a pseudoaneurysm (asterisk) (HU 283) originating from the right coronary cusp (RCC). An oblique in-plane view (Figure 6C) demonstrated both the pseudoaneurysm (asterisk) originating from the RCC and the fistulous connection (arrow) between the NCC and RA. Preceding TTE images in the apical 5-chamber view (Figure 6D) demonstrated a likely communication between the aortic valve and LVOT. Blood flow through this communication was documented with both apical 5-chamber colour Doppler (Figure 6E) and subcostal 4-chamber pulsed wave Doppler (Figure 6F). A subsequent CT was performed to delineate the fistulous connections more definitely. The CT scan also confirmed a small anterior aortic root pseudoaneurysm that had not been suspected on the TTE.

### Case 7

A 69-year-old man presented with decompensated heart failure. Blood cultures were negative. Cardiac CT 3D reconstructed images demonstrated a large and almost circumferential pseudoaneurysm (Figure 7A) (arrows) which originated from the aortic valve left coronary cusp (LCC) adjacent to the origin of left main stem coronary artery (Figures 7B and 7C). Preceding TOE images in the ME AV SAX view (Figure 7D) had shown dehiscence of the surgical bioprosthetic valve with a large pseudoaneurysm that appeared to be communicating with the LCC. The CT scan in this instance had been requested to evaluate not only the coronary arteries prior to cardiac surgery but also establish the extent of the pseudoaneurysm prior to surgical correction.

## Discussion

The 2023 guidelines for IE have placed an increased emphasis on cardiac imaging to establish a diagnosis of “definite” endocarditis. In the current manuscript we detail the successive changes to the Duke criteria over the last 30 years. We also provide validation for the incorporation of cardiac CT into current guidelines by using a spectrum of case examples from a large endocarditis centre in Europe.

Currently echocardiography is the guideline endorsed principal imaging modality utilised for the diagnosis of IE and its local complications. When the clinical suspicion for IE is low, TTE is recommended as the first line investigation with a negative study being a prompt to consider other clinical entities. When TTE image quality is likely to be low, and the clinical suspicion of IE or its complications is high (PHV, ICED, new atrio-ventricular block), TOE should be considered a first line test. In these instances, a negative TTE may be unable to rule out IE and associated local paravalvular complications (12). In this setting it remains the gold standard technique for the detection of vegetations on native heart valves where is it also permits an assessment of valve morphology (including perforation) and function without the use of ionizing radiation or iodinated contrast material. Transoesophageal echocardiography is however an invasive procedure that is not without risk (13). It is operator dependent, has a limited field of view, is subject to prosthetic valve and CIED artefacts and lacks the ability to differentiate tissue characteristics compared with alternative imaging modalities such as CT. Furthermore, echocardiography does not provide any information with regards to the potential extracardiac complications of IE (Table 2).

**Table 2:** Overview of strengths and weakness of computed tomography (CT) and transoesophageal echocardiography (TOE).

	Cardiac CT	TOE
Strengths	- Less invasive procedure - Better diagnostic accuracy of prosthetic heart valves and intra-cardiac devices - Better diagnostic accuracy for paravalvular complications - Evaluation of extracardiac emboli - Pre-operative planning - Evaluation of co-existent coronary artery disease	- No ionizing radiation - No iodinated contrast agent - Enables assessment of valvular structure and function (regurgitation, stenosis)
Weaknesses	- Ionizing radiation - Iodinated contrast agent - Nephrotoxic agent - Less diagnostic accuracy for small vegetations - Potential for hard beam artifacts with prosthetic heart valves and intra-cardiac devices - Reliant on optimal heart rate control	- Invasive procedure - No tissue characterisation - Less accurate for assessment of paravalvular complications - Potential suboptimal diagnostic accuracy of prosthetic heart valves and intra-cardiac devices - Operator dependent

This has led clinicians to seek complementary imaging techniques to improve the detection of endocarditis related intracardiac and extracardiac disease. In this regard, cardiac CT has emerged as a viable contender. While multidetector cardiac CT is not as accurate in detecting vegetations <10 mm and therefore has a reduced detection compared with TOE (sensitivity 71.4%, specificity 100%), CT is arguably superior in the detection of vegetations on prosthetic heart valves where echocardiographic evaluation may be limited as a result of mechanical discs, leaflets and stent frame artefact (14–17). Furthermore, cardiac CT is superior to TOE for the detection of paravalvular involvement with IE (sensitivity 100%, specificity 100%). This includes the detection of aortic root abscesses, pseudoaneurysms (sensitivity 100%, specificity 87.5%), intra-cardiac fistulae and valve dehiscence (14, 18) (Cases 1, 4, 6 and 7). Importantly, in NVE cardiac CT has a diagnostic accuracy of 96% (sensitivity 97%, specificity 88%) in predicting intra-operative findings (14). Based on these data, the authors endorse the use of cardiac CT as having a primary role in NVE and PVE when TOE is inconclusive or unavailable and paravalvular complications are suspected. This is in keeping with the findings of numerous other studies showing a higher detection rate of paravalvular complications in patients with PVE with CT compared to CT (18–23).

The diagnostic yield of ^18^F-FDG PET/CT is variable. In patients with suspected NVE ^18^F-FDG PET/CT performs poorly as the metabolic activity within vegetations is low and results in insufficient tracer uptake. In contrast, its value is much higher in PVE where perivalvular abscesses with increased tracer uptake are much more frequent (24). Indeed, incorporation of ^18^F-FDG PET/CT findings into the modified Duke criteria increases their sensitivity from 52%–70% to 91%–97% (class IB indication for use) (5, 25). Detection of ICED IE utilizing ^18^F-FDG PET/CT is highest for pocket size infection (sensitivity 86.7% and specificity 100%) but decreases significantly for overall ICED IE (sensitivity 30.8%, specificity 62.5%) (26). Importantly, whole body ^18^F-FDG PET/CT is particularly useful in patients with a suspicion or proven IE to identify distant lesions and mycotic aneurysms or to add a minor diagnostic criterion (27). In keeping with this data, our institutional practice is to utilize ^18^F-FDG PET/CT particularly in patients with possible PHV or pocket ICED IE (5).

Given the propensity for IE to manifest with systemic complications related to septic emboli, our practice also has evolved to include systematic whole-body and brain CT to detect systemic complications of IE. This includes septic emboli and mycotic aneurysms throughout the vascular tree, including the pulmonary (Case 5) and central nervous systems (28, 29). With neurological complications occurring in up to 30% of cases of IE, prompt utilisation of cross-sectional imaging enables earlier access to neurology, neurosurgical intervention and better guidance to the optimal timing of surgical intervention. Although CT is inferior to magnetic resonance imaging (MRI) for the detection of ischaemic and haemorrhagic neurological complications of IE (30), CT is often more feasible in critically ill patients where it exhibits a sensitivity of 90% and specificity of 86% (31). However, when further confirmation or evaluation is required, there should be a low threshold for brain MRI. Finally, CT can also detect the extra cardiac sources of the bacteraemia responsible for IE. This includes early neoplastic lesions, which may require treatment prior to potential heart valve surgery.

One should also consider cardiac CT as the preferred technique (class IB recommendation) for the evaluation of coronary arteries in patients with aortic valve IE and aortic root pathology in whom surgery is being contemplated. Not only does CT reduce the risk of catheter induced aortic injury and systemic embolization of aortic valve vegetations (6, 15, 22, 32, 33), but these findings assist with surgical planning. Knowledge of coronary anatomy, the presence of co-existent atherosclerotic coronary disease and proximity of coronary arteries to mycotic aneurysms and abscesses is critical to the surgical technique (Case 7). The added information derived from CT pertaining to the morphology, size and calcification of the aortic valve, aortic root and ascending aorta is also important. The presence of a “porcelain aorta” may preclude surgery for aortic valve IE altogether. Similarly, heavy mitral annular calcification may exclude a surgical option for mitral valve IE due to a prohibitive operative risk (34).

Further benefits of cardiac CT include its non-invasive nature and its independence of patient characteristics. Cardiac CT is largely operator independent, and at specialist centres, its ready availability often allows easier coordination and planning than TOE. In this setting, expeditious use of CT often facilitates a quicker confirmation of “definite endocarditis”. This can lead to earlier guideline-indicated cardiac surgical intervention with an anticipated reduction in mortality of approximately 50% (35). The authors endorse the current ESC guidelines for endocarditis and advocate a role for cardiac CT in routine clinical use for suspected IE when TOE is not readily available or is contraindicated/inconclusive. We also support the use of CT in all cases of suspected or confirmed PVE to detect paravalvular and systemic complications and in patients being planned for surgical intervention. Finally in patients with aortic valve endocarditis (native or prosthetic) coronary CT angiography would be considered our technique of choice for coronary evaluation prior to cardiac surgery.

Despite the advantages that cardiac CT offers in patients with suspected or confirmed IE, there are qualifications, challenges and limitations to its routine application which must be considered. Careful imaging needs to be performed to ensure optimal image quality. This includes ECG gating of images and ensuring appropriate scan protocols are selected with the appropriate exposure settings and contrast regimes. Although, ideally cardiac imaging should be performed at heart rates <60 BPM with the use of beta-blockers and GTN, this is not always viable in critically unwell patients where cardiogenic shock, severe valve regurgitation/obstruction and heart block often coexist (36). In these instances, ECG-gated multiphase cardiac CT may be used in patients with elevated heart rates or cardiac arrythmias. In patients who have an inability to breath hold for the required duration of the scan, fast acquisition protocols with a high pitched prospective ECG-gated helical scan mode may also be beneficial (37). Although this is conventionally only available on dual source CT scanners, there is promise that single source CT with artificial intelligence-based reconstruction algorithms may in turn provide similar results. The use of cardiac CT may also be challenging in patients with significant renal dysfunction where iodinated contrast agents may impact upon an existing diminished renal reserve. It should also be recognized that the presence of PHVs and ICEDs may also result in hard beam artefact that obscures the evaluation of critical structures. To overcome these issues, metallic object artefact reduction reconstruction algorithms should be used where possible. Above all, it is imperative that cardiologists and radiologists accrue the relevant experience in evaluating IE cardiac CT. A common and standardised language should be used for clinical teams.

## Conclusions

Current guidelines recommend that IE care should be coordinated within regions by a dedicated team that resides at a specialist centre (6). This “Infective Endocarditis” team should be comprised of infectious disease specialists, cardiologists with a specialist interest in heart valve disease/cardiac imaging, cardiac surgeons, and cardiac device specialists. With the recent 2023 ESC IE Guidelines reinforcing the value of advanced imaging (6), specialist centres treating IE should also have ready access to cardiac CT, MRI and ^18^F-FDG-PET alongside their existing ultrasound modalities (38). Although these modalities are often available, their clinical use is likely variable from institution to institution. While we await further validation of the new guidelines, our experience with the systematic incorporation of early cardiac CT endorses current recommendations whilst recognising the strengths and weaknesses of each imaging modality. Cardiac CT ultimately offers the potential to “bridge the detection gap” between “possible” and “definite” IE. By achieving an earlier recognition of IE and both its cardiac and extracardiac complications we anticipate that this will result in earlier timely referrals for cardiac surgery and ultimately improved clinical outcomes.
